# Recurrent severe invasive pneumococcal disease in an adult with previously unknown hyposplenia

**DOI:** 10.1186/s12879-015-0883-2

**Published:** 2015-04-02

**Authors:** Vibe C Ballegaard, Lone Schejbel, Steen Hoffmann, Bjørn Kantsø, Christian P Fischer

**Affiliations:** Department of Infectious Diseases, Copenhagen University Hospital, Blegdamsvej 9, DK 2100 Rigshospitalet, Denmark; Department of Clinical Immunology, Copenhagen University Hospital, Rigshospitalet, Denmark; Department of Microbiology & Infection Control, Statens Serum Institute, Copenhagen, Denmark; Department of Microbiological Diagnostics & Virology, Statens Serum Institute, Copenhagen, Denmark

**Keywords:** Hyposplenia, Immunodeficiency, Invasive pneumococcal disease, Vaccination

## Abstract

**Background:**

The risk of life-threatening and invasive infections with encapsulated bacteria is increased in patients with hyposplenia or asplenia. We report a case of recurrent invasive pneumococcal meningitis in a woman with previous unknown hyposplenia. She was vaccinated after the first episode of meningitis and developed sufficient levels of pneumococcal antibodies. The pneumococcal strains isolated were serotype 7 F and 17 F. To our knowledge, there has been no previously reported case of recurrent invasive pneumococcal disease in a pneumococcal vaccinated adult with hyposplenia and apparently sufficient antibody response.

**Case presentation:**

We report the course of a 38-year-old Caucasian woman presenting with recurrent episodes of invasive pneumococcal disease (IPD) and previously unknown hyposplenia. Hyposplenia was discovered during the second episode of IPD and no underlying medical condition was found. Despite immunization against *S. pneumoniae* and measurement of what was interpreted as protective levels of serotype-specific IgG antibodies after vaccination, the patient suffered from a third episode of IPD.

**Conclusions:**

Individuals with predisposing medical conditions or a history of severe infections with encapsulated bacteria should be screened for spleen dysfunction. If splenic function is impaired, prevention against severe invasive infection with encapsulated bacteria are a major priority.

## Background

*Streptococcus pneumoniae* may cause severe invasive pneumococcal disease (IPD) which includes pneumonia, sepsis and meningitis [1]. The spleen plays important roles in antigen processing. Initiation of an immunological response, production of specific antibodies and splenic macrophages are important for removal of encapsulated bacteria and intra-erythrocytic parasites. Thus, hyposplenic or asplenic patients are at increased risk for fulminant and life-threatening infections with encapsulated bacteria, most likely due to lack of splenic filtering and decreased production of specific antibodies and memory B lymphocytes [2,3]. Patients with hyposplenia or asplenia have reduced levels of IgM memory B cells and IgM anti-pneumococcal antibodies, causing reduced ability to produce protective antibodies against polysaccharide antigens and hence possible vaccine failure [2,4,5]. The risk of severe invasive infections with encapsulated bacteria in splenectomised patients is more than 50-times higher than in the general population, and 50-90% of the cases are caused by *S. pneumoniae*, followed by *H. influenzae* and *N. meningitidis* [6].

Asplenia refers to the absence of the spleen, a rare congenital disorder and most often a result of surgery. Hyposplenia is regarded as an acquired disorder associated with several medical diseases, and sometimes accompanied by a reduction in spleen size. The most frequent hyposplenia-associated disorders are severe liver disease, ulcerative colitis, celiac sprue, and lupus. Hemoglobinopathies, such as sickle cell disease and the thalassemia’s, are also important causes of functional hyposplenia [2]. Prevention of IPD in patients with hyposplenia includes education, vaccination and antibiotic prophylaxis [7]. However, IPD can occur even in patients who have received pneumococcal immunization and antibiotic prophylaxis.

To our knowledge, this is the first reported case of recurrent IPD in a pneumococcal vaccinated adult with hyposplenia and an estimated sufficient antibody response. Collection of data was performed according to guidelines from the Danish Data Protection Agency and the local ethics committee and in accordance with the Declaration of Helsinki.

## Case presentation

### First episode of IPD

A 38-year-old Caucasian woman was hospitalized in 2008 with IPD (serotype 7 F) causing meningitis, septic shock and disseminated intravascular coagulation (DIC). Sequelae included total hearing loss treated with bilateral cochlear implants and amputation of the four lateral toes of the left foot. Hereditary immunodeficiency was suspected, as a one-year-old daughter was hospitalized with pneumococcal meningitis (serotype 7 F) one month earlier. Immunological investigation of the patient and her daughter included assessment of complement activation, fraction and concentrations of T, B and NK lymphocytes and subpopulations, proliferative response of CD4 T-cells, and Toll-like-receptor-stimulation. No signs of immunodeficiency were found. The only abnormal finding was a low level of IgM-positive CD27 memory B cells in the mother. The child was too young for assessment of memory B cells. No screening for impaired spleen function was made at that time. The patient was immunized with a 23-valent pneumococcal polysaccharide vaccine (PPV23, Pneumovax®, Sanofi Pasteur MSD) one month after admission, but measurement of the vaccination response was not performed. Retrospective analysis of serum IgG-antibody-levels against 14 representative serotypes was performed on samples taken 5 days and 20 days after debut (Table 1). A cut-off level of at least 0.35 μg/mL was estimated as a protective level against IPD. Antibody levels against the serotypes 3, 4, 5 and 7 F were below the cut-off level of at least 0.35 μg/mL and the patient continued to have apparently not protective levels of antibodies against serotype 7 F despite the current infection. The cut-off level of 0.35 μg/mL is recommended by a WHO Working Group as applicable for assessing the efficacy of pneumococcal conjugate vaccines and is widely considered to provide a protective effect against IPD [8]. However, it should be noted that this recommendation relates to measurement of antibody-levels after vaccination with the conjugate vaccines and is based on trials that analyses protective levels in infants after vaccination [8].

**Table 1:** **Serotype-specific** ***Streptococcus pneumoniae*** **Immunoglobulin G titres (IgG) in an adult with previously unknown hyposplenia suffering from recurrent episodes of invasive pneumococcal disease (IPD)**

**IPD event, serotype**	**Date**	**Serotype,** **μg/ml #**													
		**1**	**3**	**4**	**5**	**6B**	**7F**	**9V**	**12F MFI**	**14**	**17F MFI**	**18C**	**19A**	**19F**	**23F**
**1** ^**st**^ **IPD, day 5, 7F**	17.08.2008	0.91	**0.09**	**0.20**	**<0.02**	0.58	**0.13**	0.47	239.5	2.97	281,3	1.06	0.67	0.85	1.72
**1** ^**st**^ **IPD, day 20, 7F**	02.09.2008	1.00	**0.09**	**0.23**	**<0.02**	0.59	**0.17**	0.50	250.3	2.73	295.5	0.86	0.77	1.55	1.75
**2** ^**nd**^ **IPD, day 16, 17F**	29.07.2012	2.81	0.38	0.49	0.94	2.54	1.23	3.07	387.8	3.49	367.5	2.58	3.14	3.97	5.35
**PCV13 immunization**	13.09.2012	**	**	**	**	**	**	**		**		**	**	**	**
Post-immunization*	30.10.2012	5.62	0.57	**0.21**	0.41	6.02	0.42	5.02	*n/a*	33.24	*n/a*	12.32	1.99	2.67	2.48
**3** ^**rd**^ **IPD, day 3, 17F**	06.01.2013	3.23	**0.20**	**0.12**	**0.18**	2.00	**0.18**	2.05	174.5	15.52	198.5	3.84	0.82	0.97	1.31

#, Blood samples were analyzed for antigen-specific IgG-titers against 14 different pneumococcal serotypes measured by a Luminex xMAP microsphere-based liquid array modified from Pickering et al. [Pickering JW, Hill HR. Measurement of antibodies to pneumococcal polysaccharides with luminex xMAP microsphere-based liquid arrays. Methods Mol Biol. 2012;808:361–75]. The type-specific protective IgG-titers are unknown. Levels beneath 0.35 μg/ml are marked with bold as studies suggest that titers above 0.35 μg/ml provide protection against invasive pneumococcal disease after vaccination with a conjugated vaccine. IgG-titers are listed in μg/ml based on the 89SF international standard, except 12 F and 17 F, which are listed in mean fluorescence intensity (MFI) because a reference material were not available.

*Average of two measurements on the same sample.

**, Serotypes covered by the PCV13 vaccine.

*n/a*, not available.

### Second episode of IPD

**Figure 1:**
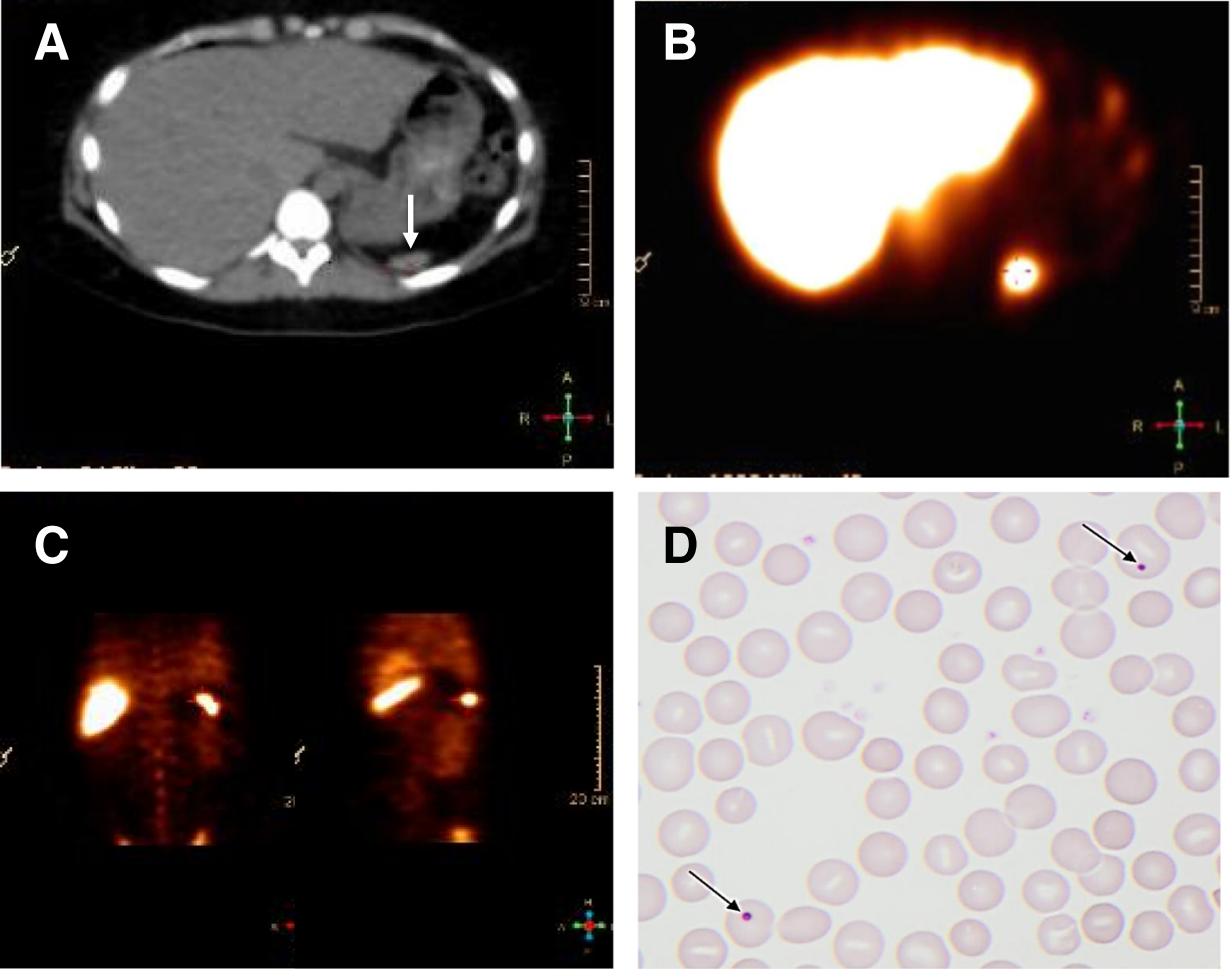
**CT scan (A), spleen scintigraphy (B, C) and peripheral blood smear (D) of a woman with recurrent episodes of invasive infections with** ***Streptococcus pneumoniae*** **. A**: CT scan showing horizontal section of a normal liver, while a very small rudimentary spleen is located at the tip of the arrow. **B**: Scintigraphy after injection of Technetium-99 m (99mTc) labeled, denatured red blood cells (RBC). The arrow shows uptake corresponding to the rudimentary spleen seen with the CT scan. **C**: Tc99m labeled, denatured RBC scintigraphy with different coronal sections of the abdomen showing the uptake corresponding to the rudimentary spleen seen with the CT scan. **D**: Peripheral blood smear stained with a standard H&E staining. Arrows point at red blood cells with Howell-Jolly bodies (HJB).

In 2012, the patient was admitted with fever, neck stiffness, confusion, septic shock, DIC and peripheral tissue ischemia. Lumbar puncture revealed cerebrospinal fluid (CSF) with 287 · 10^6^/L leukocytes of which 189 · 10^6^/L were neutrophils. Cultures of CSF and blood were positive for *S. pneumoniae* and later serotyping showed type 17 F. On admission, blood laboratory analyses showed: leucocyte count 43 · 10^9^/L, platelet count 35 · 10^9^/L, hemoglobin 6.4 mmol/L, C-reactive protein 300 mg/L, creatinine 245 μmol/L, alanine aminotransferase 730 U/L, lactate dehydrogenase 2980 U/L, D-dimer 138 mg/L, PP 0.2, INR 2.4, antithrombin 0.42 kIU/l, and fibrinogen 3.1 μmol/L. Initial empirical treatment for bacterial meningitis was intravenous ceftriaxone, ampicillin and dexamethasone. On day 2, the *S. pneumoniae* strain was found to be fully sensitive to penicillin, and treatment was changed to high-dose penicillin G. Infusion of epoprostenol was administered due to severe peripheral ischemia. Measurement of plasma immunoglobulin revealed low IgM at 0.17 g/L, but normal IgG and IgA, and the patient was treated with single intravenous infusions of immunoglobulin (IVIG) at day 3 to day 5. The cochlear implant was suspected to be the infectious focus, but CT scan of the head with intravenous contrast showed no signs of infection. Transthoracic and trans-esophageal echocardiography revealed no signs of endocarditis or cardiovascular abnormalities and the chest X-ray showed no infiltrates. An ultrasound investigation of the abdomen was performed, during which no spleen could be detected, and a subsequent CT scan of the abdomen and thorax with intravenous contrast revealed a small irregularly defined and partially calcified mass measuring 3.8 × 2.1 cm interpreted as a small spleen (Figure 1A). No enlarged lymph nodes were detected and all other organs were unremarkable. A Technetium-99 m (^99m^Tc) labelled heat-denatured red blood cell scintigraphy showed a small rudimentary spleen (2 cm) at the same location as with the CT scan (Figure 1B,C), and a blood smear revealed Howell-Jolly bodies (HJB), indicating an impaired function of the spleen (Figure 1D). No genetic defects were found by analysis of chromosomes and comparative genomic hybridization array. The patient recovered to normal consciousness within day 1 and was discharged on day 16 after two weeks of treatment with high-dose penicillin G. Persistent mild cognitive impairment was the only apparent sequelae. On discharge, ‘stand-by’ antibiotics (amoxicillin/clavulanic acid) for use in case of fever were provided.

IgG-antibody-levels against the 14 pneumococcal serotypes were examined 16 days after admission (Table 1), and the results suggested protective titers against all 14 serotypes, however, probably influenced by the recent immunoglobulin infusion. The patient was vaccinated with the 13-valent pneumococcal conjugate vaccine (PCV13, Prevenar 13®, Pfizer) and with the *Haemophilus influenzae* type b (ACT-HIB®, Sanofi Pasteur MSD) vaccine two months after debut. IgG antibody-levels were evaluated one and a half month after vaccination, showing decreasing average antibody levels from 5.48 μg/mL to 4.42 μg/mL although levels beneath 0.35 μg/mL were only found against serotype 4 (Table 1).

The patient has three children, at that time aged 2 (M), 4 (F) and 7 (F) years. All three children were healthy with no obvious signs of immunodeficiency. The children were examined with abdominal ultrasound in order to detect a possible hereditary asplenia or hyposplenia. The abdominal ultrasound was normal, except from the finding of a slightly small spleen in the 4-year old daughter that as 1-year old was hospitalized with pneumococcal meningitis (serotype 7 F). The children were not evaluated for Howell-Jolly bodies. All children had normal immunoglobulin levels including IgG-subtypes and normal response to vaccination against tetanus, diphtheria and *H. influenzae* type b. All the children were vaccinated with the PCV13 vaccine.

### Third episode of IPD

**Figure 2:**
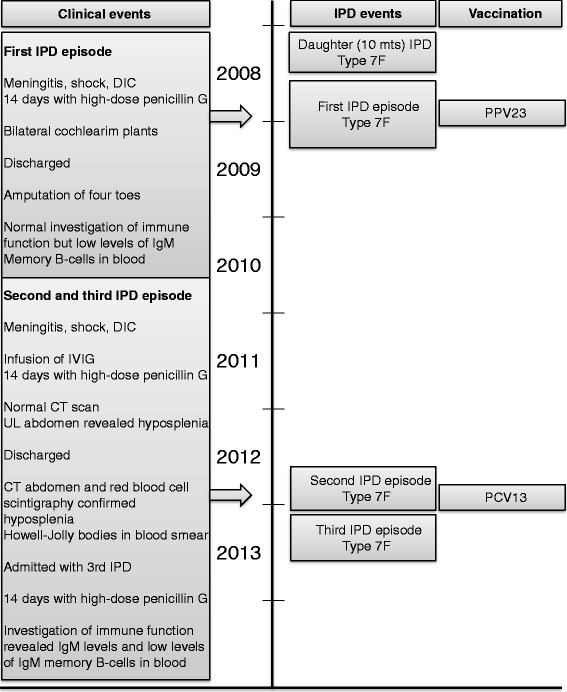
**Timeline of IPD episodes and clinical events.**

The patient was admitted with a third episode of IPD five and a half month after the second episode. The patient took stand-by antibiotics prior to admission, but due to vomiting probably without effect. The symptoms started less than 24 hours before admission on which the patient presented with septic shock but without signs of meningitis or neurological dysfunction. Lumbar puncture was performed and CSF contained 12 · 10^6^/L leukocytes of which 2 · 10^6^/L were neutrophils. Again, *S. pneumoniae* was cultured in blood, but also detected in CSF using PCR with specific primers for *S. pneumoniae*. Typing showed infection with serotype 17 F. The *S. pneumoniae* strain was fully sensitive to penicillin, and accordingly the patient was treated with high-dose penicillin G for two weeks. The patient rapidly recovered without new sequelae. Detailed reassessment of the immune function was performed one month after recovery. A mild lymphocytosis with increased concentrations of B cells, increased concentrations of CD8 T cells , normal fractions of T, B and NK cells, normal expression of CD45RA/CD45RO among T cells, but a reduced level of IgM positive memory B cells and reduced levels of IgM in blood was found. The proliferative response of CD4 T-cells to stimulation with CD3/CD28/CD2 or pokeweed mitogen was within the normal range. In serum day 3 after admission IgG-pneumococcal-antibody-levels against the 14 serotypes were measured. It applied to all subtypes that antibody-levels declined since the post-immunization measurement or regarding 17 F and 12 F since measurements from the first two episodes of IPD. However, only antibody-levels against 3, 4, 5 and 7 F were below the cut-off level of 0.35 μg/mL. Information from the patient’s history organized as a timeline is summarized in Figure 2.

## Conclusions

The patient presented above had no history suggesting immunodeficiency until the first episode of IPD. However, when interviewed after the second episode of IPD, she recalled having had pneumonia about once a year from about the age of 18 treated by her general practitioner. Additional information and verification of infectious episodes could not be obtained. A predisposing medical disease associated to hyposplenia was not suspected or found. Examination of splenic dysfunction is rarely performed routinely in first-time meningitis unless there is a specific suspicion. Spleen dysfunction was not suspected in this patient although the immunological investigation demonstrated a low level of IgM-positive CD27 memory B cells, which could represent a sign of spleen dysfunction. Establishing the diagnosis of functional hyposplenia is based on the spleen filtering function of radiolabelled isotopes or by detection of morphological erythrocyte abnormalities. Erythrocyte scintigraphy is currently the best approach for evaluating functional hyposplenism [9], although usually not available as a routine investigation. Examination of peripheral blood for numbers of pitted erythrocytes (erythrocytes with characteristic membrane pits) has proven to be a sensitive method, which accurately correlates with spleen volume, and is therefore used as gold standard for spleen function in many studies [10]. However, the method is not widely used, and requires specific equipment. A more commonly used method is observation of erythrocytes with nuclear remnants named Howell-Jolly bodies (HJB) on blood smears. Detection of HJB is a useful method of screening for asplenia, although the specificity and sensitivity has been disputed, especially regarding cases with mild hyposplenia [11–13]. Flowcytometric quantification of HJB is also a useful method [14]. Reduction of spleen size using ultrasound or computed tomography can be used to detect hyposplenia, but it is unclear whether non-functional imaging reveals useful information about risk for severe infections or spleen function [15].

The patient was vaccinated with the PPV23 vaccine after the first episode of IPD. However, the antibody response was never evaluated. After the second episode of IPD, the antibody response against PPV23 was measured using a calculated geometrical mean of 12 representative capsule-types (1, 3, 4, 5, 6B, 7 F, 9 V, 14, 18C, 19A, 19 F), and interpreted as sufficient. It should be noted that the patient during the second episode of IPD received treatment with immunoglobulin and evaluation of protective antibodies did perhaps not reflect the vaccination response, but rather protection by specific immunoglobulin’s administered during hospitalization. Eight weeks after the second episode of IPD, the patient received a dose of PCV13. Antibody titers against the 12 representative capsule-types were evaluated and found to be above the cut-off indicating protection. As suggested by guidelines, the vaccination was not followed by PPV23, as the patient received the first dose of PPV23 four years earlier.

Guidelines by the Advisory Committee on Immunization Practices (ACIP) recommend prophylactic vaccination against *S. pneumoniae*, *H. influenzae* type b and *N. meningitis* in patients with spleen dysfunction [12]. Vaccination at the first opportunity is recommended, and should be followed by re-immunizations every 5 years [7]. The ACIP guidelines ) has recently been changed for adults > 19 years old with immune-compromising conditions due to increasing evidence of immunological hypo-responsiveness induced by prior vaccination with PPV23 and reduced effectiveness of PPV23 [16,17]. A primary combined schedule of PCV13 (serotypes 1, 3, 4, 5, 6A, 6B, 7 F, 9 V, 14, 18C, 19A, 19 F, and 23 F) followed by PPV23 (serotypes 1, 2, 3, 4, 5, 6B, 7 F, 8, 9 N, 9 V, 10A, 11A,12 F, 14, 15B, 17 F, 18C, 19 F, 19A, 20, 22 F, 23 F, and 33 F) eight weeks later, and an additional PPV23 five years later is now recommended [18]. If at least one dose of PPV23 has been administered previously, a dose of PCV13 is recommended one year after the last PPV23 dose. For those who require additional doses of PPV23, the first such dose should be given no sooner than 8 weeks after PCV13 and at least 5 years after the most recent dose of PPV23 (Table 2) [18]. The combined use of the heptavalent conjugate vaccine (PCV7, serotypes 4, 6B, 9 V, 14, 18C, 19 F, and 23 F) followed by the PPV23 vaccine has proven efficient in hyposplenic patients [19,20], but to our knowledge the clinical efficacy of PCV13 and PPV23 in combination has not yet been evaluated in hyposplenic patients.

**Table 2:** **Recommended immunizations against invasive pneumococcal disease using the 13-valent pneumococcal conjugate vaccine (PCV13) and the 23-valent pneumococcal polysaccharide vaccine (PPV23) for adults aged ≥19 years with functional or anatomic asplenia [** 17]

**Vaccination status**	**PCV13**	**PPV23**	**Antibody measurement**
Pneumococcal vaccine-naïve	1 dose of PCV13	1 dose of PPV23 ≥ 8 weeks after PCV13	Prior to revaccination with PPV23
		Revaccination with PPV23 ≥ 5 years after last PPV23	
Earlier vaccinated with PPV23	1 dose of PCV13 ≥ 1 year after last PPV23	Revaccination with PPV23 ≥ 8 weeks after PCV13 and ≥ 5 years after last PPV23	Prior to revaccination with PPV23

The presented case emphasizes that even after vaccination and when serotype-specific IgG responses seems to be protective, hyposplenic patients can be vulnerable to IPD. Currently, measurement of IgG-pneumococcal antibodies does not seem very useful for determining the degree of protection against IPD in hyposplenic patients, and no studies have established the necessary protective levels. Moreover, decreased quality of the antibodies and inefficient cellular immune responses could probably have a greater impact on the risk of IPD than the actual amount of antibody in this patient group. Measurement of specific memory B cells and functional antibodies (opsonophagocytic activity, OPA) could further elucidate a possible hypo-responsiveness, but these studies are not available in our laboratory, nor are commonly used methods for assessing vaccination response in most clinical settings.

In this case the patient received a dose of PPV23 at the first episode of IPD. Thus, the possibility of PPV23-induced immunological hypo-responsiveness upon re-vaccination has to be considered. A recent study demonstrated impaired antigen-specific B-cell responses in asplenic patients with β-Thalassemia previously vaccinated with PPV23. Antigen-specific memory B-cells and IgG-pneumococcal antibodies were measured, and a time- and dose-dependent negative effect of previous PPV23 vaccination was detected [21].

Yearly administration of influenza vaccination is recommended, because it reduces the risk of secondary pneumococcal and *H. influenzae* infection [7]. A recent study showed a 53% reduced risk of death in asplenic persons who received influenza immunization compared to those who had no influenza immunization. Interestingly, no influence on death was seen for pneumococcal immunization [22].

In patients who fail to have an adequate response to pneumococcal vaccine it has been proposed that lifelong antibiotic prophylaxis could be considered [23]. The use of antibiotics for the prevention of IPD is not evidence based, and differs between guidelines. Many guidelines recommend prophylaxis with 250–500 mg per day of amoxicillin or 500 mg per day of phenoxymethylpenicillin [7,12,24,25]. No consensus regarding duration of prophylaxis exists. British guidelines propose lifelong treatment. Another approach is access to ‘stand-by’ antibiotics. ‘Stand-by’ antibiotics should be taken at the first sign of infection if the patient is unable to obtain prompt medical attention. A strategy with 2 years of prophylactic antibiotics followed by the use of emergency or standby antibiotics and early presentation to medical care has been proposed [26]. In the present case the patient received stand-by antibiotics after the second episode of IPD and had a good knowledge of the risk of severe infections, but nevertheless suffered from another episode of IPD. However, the third episode was less severe than the first and second episode, and the patient recovered quickly. This could probably be explained by earlier initiation of treatment with intravenous antibiotics.

Individuals with predisposing medical conditions and a history of invasive infections with encapsulated bacteria’s or recurrent episodes of invasive infections should be referred to a specialist unit with expert knowledge in infectious diseases and with the ability to perform screening for spleen dysfunction assessed by erythrocyte scintigraphy, pitted erythrocyte counting, or Howell-Jolly body detection. If splenic function is reduced, prevention against infection is a major priority. Prophylactic initiatives should include immunization strategies in accordance with current guidelines supplemented by stand-by antibiotics and education of patients and professionals. Sufficient levels of serotype-specific IgG antibodies do not exclude the possibility of new episodes of IPD.

## Consent

Written informed consent was obtained from the patient for publication of this Case report and any accompanying images. A copy of the written consent is available for review by the Editor of this journal.

## Abbreviations

CSF: Cerebrospinal fluid
CT scan: Computed tomography
DIC: Disseminated intravascular coagulation
*H. influenza*: *Haemophilus influenzae*
HJB: Howell-jolly bodies
IgG: Immunoglobulin G
IgM: Immunoglobulin M
IPD: Invasive pneumococcal disease
IVIG: Intravenous infusions of immunoglobulin
*N. meningitides*: *Neisseria meningitidis*
PCV13: 13-valent pneumococcal conjugate vaccine
PPV23: 23-valent pneumococcal polysaccharide vaccine
*S. pneumonia*: *Streptococcus pneumoniae*
